# Favorable long-term outcomes of autoimmune nodopathy with mycophenolate mofetil

**DOI:** 10.3389/fneur.2024.1515161

**Published:** 2024-12-12

**Authors:** Young Gi Min, Woohee Ju, Jung-Joon Sung

**Affiliations:** ^1^Department of Translational Medicine, Seoul National University College of Medicine, Seoul, Republic of Korea; ^2^Department of Neurology, Chung-Ang University Hospital, Seoul, Republic of Korea; ^3^Department of Neurology, Seoul National University Hospital, Seoul, Republic of Korea; ^4^Neuroscience Research Institute, Seoul National University College of Medicine, Seoul, Republic of Korea; ^5^Wide River Institute of Immunology, Seoul National University, Hongcheon, Republic of Korea

**Keywords:** autoimmune nodopathy, immunotherapy, outcomes, autoantibody, electrophysiology

## Abstract

Autoimmune nodopathy (AN) is a rare immune-mediated neuropathy characterized by autoantibodies against nodal or paranodal proteins. Patients with AN generally respond poorly to immunoglobulin therapy, and as a newly defined condition, there are currently no established treatment guidelines. Although rituximab shows potential as a therapeutic option, its high cost, limited availability, and the need for infusion monitoring hinder its use as a first-line treatment in many countries. In this report, we identified AN antibodies in five of 106 serum samples (4.7%) prospectively collected from patients initially diagnosed with chronic inflammatory demyelinating polyradiculoneuropathy (CIDP): anti-neurofascin 155 (NF155) in 2 patients, anti-contactin-1 (CNTN1) in 1, anti-contactin associated protein 1 (CASPR1), and anti-NF186/140 in 1. Notably, we observed favorable long-term outcomes in these patients following treatment with mycophenolate mofetil (MMF) and corticosteroids. Given that these patients had not responded to immunoglobulin therapy and/or experienced relapses with corticosteroid monotherapy in their prior episodes, we propose MMF as a cost-effective treatment strategy for AN.

## 1 Introduction

Autoimmune nodopathy (AN) is a group of immune-mediated neuropathies caused by autoantibodies against proteins at the paranodal junctions or the nodes of Ranvier (1). Four major autoantigens are known so far: neurofascin-155 (NF155), contactin-1 (CNTN1), contactin-associated protein 1 (CASPR1), and NF186. These antibodies disrupt the attachment of the terminal loops of myelin sheath to the axolemma, leading to significant conduction deficits without macrophage-mediated segmental demyelination (2). Typical clinical manifestations include sensory ataxia, distal weakness, and tremor, while patients with pan-NF antibodies may present with a fulminant form of Guillain-Barre syndrome (GBS). Intravenous immunoglobulin (IVIG), a standard option for GBS and chronic inflammatory demyelinating polyradiculoneuropathy (CIDP), is often ineffective in AN, presumably due to the predominance of IgG4 subclass. Instead, an anti-CD20 monoclonal antibody rituximab has emerged as a promising treatment option, with a clinical trial ongoing for refractory AN (3). However, its use is often limited due to the high cost or low availability in many countries including South Korea, and infusion-related challenges. Thus, there remains an unmet need for optimal treatment strategies for this novel and rare disease. Here, we describe clinical histories of 5 patients with AN identified in a prospective cohort of 106 CIDP serum samples and report their favorable long-term outcomes after mycophenolate mofetil (MMF) and corticosteroids treatment.

## 2 Methods

### 2.1 Study participants and data collection

This study is based on consecutive serum samples prospectively collected from patients with a CIDP diagnosis who visited Seoul National University Hospital (SNUH) from April 2023 to April 2024. All included patients had relevant clinical features and nerve conduction study (NCS) findings meeting demyelinating criteria based on the 2021 EAN/PNS guidelines (4). The diagnosis of AN was established by confirming samples that tested positive in live cell flow cytometry assay (see Section 2.3) with a second technique, mouse nerve immunofluorescence assay (IFA) (see Section 2.4), as recommended by the guideline. We investigated clinical presentation, NCS and cerebrospinal fluid (CSF) findings, treatment regimens and outcomes of those diagnosed with AN. Inflammatory Neuropathy Cause and Treatment (INCAT) and the CIDP disease activity status (CDAS) were employed as a long-term outcome indicating disability and disease activity, respectively (5). Any available follow-up sera were utilized to determine serial changes in antibody titers. In addition, summated compound muscle action potential (CMAP) peak-to-peak amplitudes and the number of NCS parameters meeting the demyelinating criteria from four routinely recorded motor nerves (unilateral median, ulnar, peroneal, and tibial nerves), hereafter referred to as demyelinating findings, were used as electrophysiological outcomes.

This study was conducted in accordance with the Declaration of Helsinki and was approved by the Institutional Review Board of SNUH (IRB no. 2403-095-1521). All participants provided written informed consent prior to participating in the study.

### 2.2 Treatment regimen

Treatment followed the local protocol. In South Korea, IVIG is not covered by national health insurance as first-line treatment for CIDP. Therefore, patients with CIDP at SNUH are typically started with intravenous methylprednisolone (IVMP, 1 g for 5 days) followed by oral prednisolone (PSL), beginning at 60 mg per day and maintained for about 2 months before gradually tapering off. MMF is commonly used as a steroid-sparing agent, starting at 500 mg twice a day and increasing to a maintenance dose of 1,000 mg twice a day. In cases where there was lack of clinical improvement or repeated relapses despite above treatment, IVIG was administered at 1 g/kg for 2 days or 400 mg/kg for 5 days, and re-treatment was based on the individual clinical course. Alternatively, rituximab or cyclophosphamide was attempted depending on the case.

### 2.3 Live cell flow cytometry assay

Antibody assay for AN was performed with slight modifications from previously described methods (6). Briefly, human embryonic kidney 293 T (HEK293T) cells were transfected with cDNA encoding full-length human NF155, NF186, CNTN1, or CASPR1, each of C-terminal fluorescence tags. After 48 h, the cells were detached, washed, and blocked. Subsequently, the cells were incubated with 100ul of serum diluted with flow cytometry buffer at 1:25 for 1 h. After washing, the cells were then incubated for 45 min with goat anti-human IgG Fc Alexa647 (1:2000, Jackson ImmunoResearch). After washing, 10,000 cells per sample were analyzed using a flow cytometer (BD Biosciences). The median fluorescence intensity (MFI) ratio was calculated by dividing the Alexa647 MFI of transfected cells with that of non-transfected cells. The cut-off for positive binding was calculated as the mean MFI ratio of 42 healthy controls plus 5 standard deviations (NF155: 3.31, NF186: 3.65, CNTN1: 1.87, CASPR1: 1.85). Longitudinal changes in antibody titers over time were analyzed using delta MFI values obtained from a single batch. Delta MFI was calculated by subtracting the Alexa647 MFI of non-transfected cells from that of transfected cells. IgG subclass analysis was performed using mouse anti-human IgG1, IgG2, IgG3, and IgG4 Alexa647 as secondary antibodies (1:500, Southern Biotech). In cases with positive results to NF155 or NF186, an additional binding assay was performed using a human full-length NF140 cDNA.

### 2.4 Mouse nerve IFA

Results of live cell flow cytometry assay were confirmed by mouse nerve IFA, which visually verified the binding of IgG from patient serum to the paranodes or nodes of Ranvier in peripheral nerves. Sciatic nerves were extracted after perfusing adult C57BL/6 mice. After overnight fixation, they were cut into 7-μm thick cryosections. The sections were washed with PBS and then permeabilized with 0.3% Triton-X in PBS (PBST). They were blocked using 0.3% PBST containing 5% normal goat serum (NGS). Subsequently, they were incubated overnight in 0.3% PBST containing 1% NGS, rabbit anti-NF155 monoclonal antibody (1:200, Cell Signaling Technology), and patient serum (1:200). After washing, the sections were incubated for 1 h in 0.3% PBST containing 1% NGS, goat anti-rabbit IgG Cy3 (1:1000, Jackson ImmunoResearch), and goat anti-human IgG Fc Alexa488 (1:1000, Jackson ImmunoResearch). Washed sections were imaged using a confocal microscope (LSM900, Zeiss).

### 2.5 Statistical analysis

Statistical analyses were performed using R software (R Core Team, version 4.2.1). Descriptive statistics are presented as number and percentage values for categorical variables and as mean and standard deviation or median and interquartile-range (IQR) values for continuous variables, as appropriate. The clinical course of the patients was visualized using Prism 8 (version 8.0.2).

## 3 Results

### 3.1 Identification of AN in a prospective cohort

**Figure 1:**
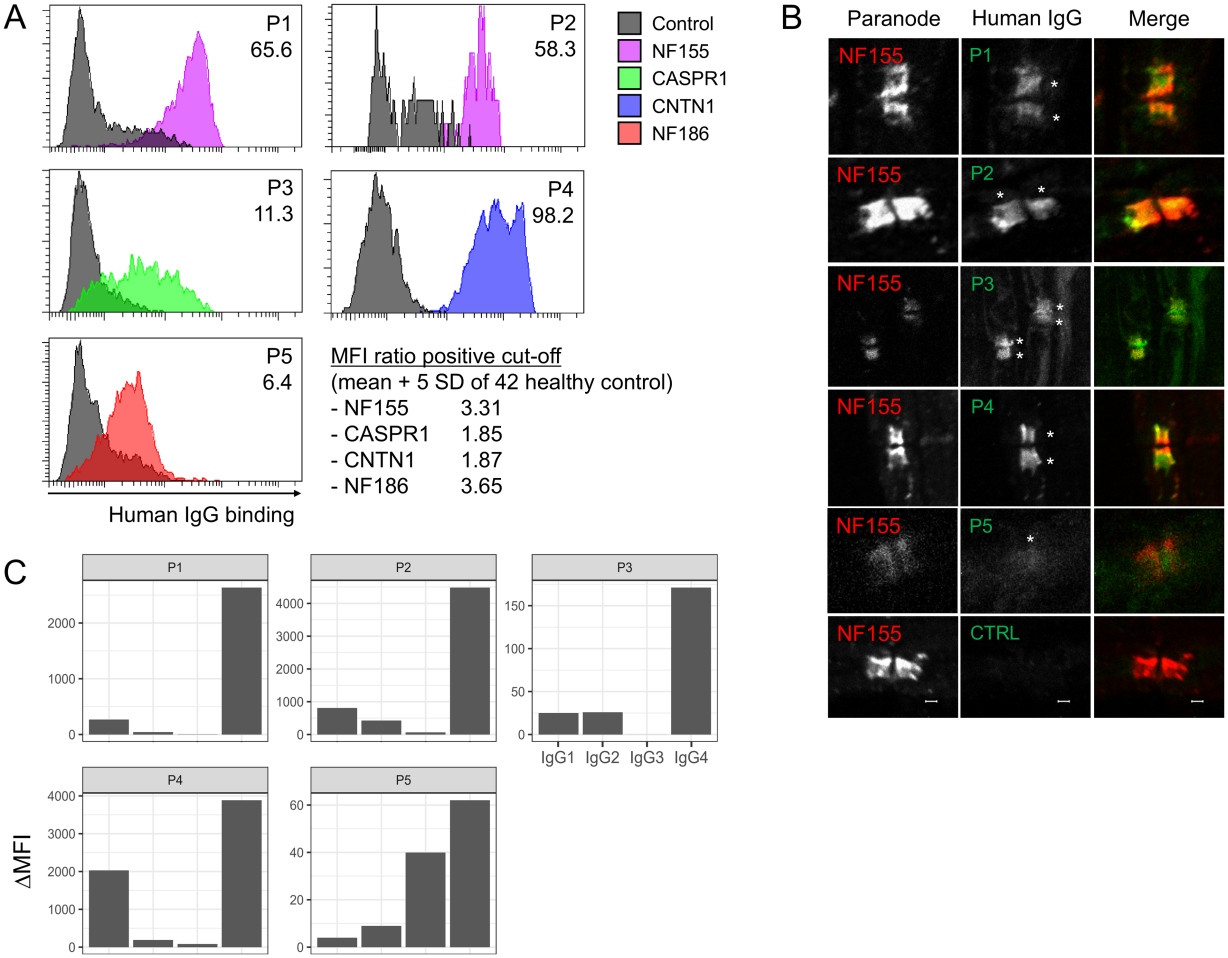
Summary of antibody assays. **(A)** Flow cytometry assay shows that patient IgG specifically binds to each type of transfected cell (colored) but not to non-transfected cells (gray). Different colors represent cells expressing different proteins. The x-axis represents the binding intensity of human IgG. The number in the upper right corner indicates the MFI ratio, of which positive cut-off was established based on data from 42 healthy controls. **(B)** Mouse nerve IFA shows that serum from P1 ~ P4 clearly binds to the paranode, while serum from P5 stains the node of Ranvier (middle column). The left column displays labeling of the paranode using a commercial antibody against NF155. The bottom row presents the results from serum of a control subject. **(C)** The antibody subclass analysis using flow cytometry demonstrates IgG4 predominance in all patients. NF155, neurofascin-155; CASPR1, contactin-associated protein 1; CNTN1, contactin-1; SD, standard deviation; CTRL, control; MFI, median fluorescence intensity; IFA, immunofluorescence assay.

The results of antibody assays are summarized in Figure 1. Among 106 sera with CIDP diagnosis, antibodies for AN were detected in 5 samples (4.7%) using the flow cytometric assay (Table 1); anti-NF155 in P1 and P2, anti-CASPR1 in P3, and anti-CNTN1 in P4. P5 tested negative for NF155 but showed positive binding to NF186 and NF140, in contrast to P1 and P2, who were specifically positive for NF155 but negative for NF186 and NF140 (Supplementary Figure S1). In mouse nerve IFA, sera from P1 to P4 demonstrated a clear binding to paranodes, while P5 serum bound to the node of Ranvier (Figure 1B). These findings align with the target locations of each patient’s autoantibodies NF155, CNTN1, and CASPR1 at the paranode, NF186 at the node, supporting the positive testing in flow cytometry assay. The predominant IgG subclass was IgG4 in all patients, with IgG1-3 detected at lower intensities in some cases (Figure 1C).

**Table 1:** Clinical, serological, and electrophysiological characteristics of patients.

Patient (antibody)	Age of onset/sex	Onset	Pre-treatment disease duration (months)	Number of previous episodes	Symptoms	Number of demyelinating findings (in 4 motor nerves)	CSF profile (WBC: /mm^3^, protein: mg/dL)	Comorbidity	Response to previous treatments
P1 (NF155)	28/M	Subacute	12	3	Distal weakness, sensory ataxia, paresthesia, hypoesthesia	DML: 3, F: 3, NCV: 2, DUR: 2, TD: 1	WBC 2 Protein 257	–	IVIG: no, IVMP+PSL: partial but relapsed
P2 (NF155)	45/F	Chronic	39	3	Sensory ataxia, tremor, distal weakness, neuropathic pain	DML: 2, F: 2, NCV: 2, TD: 2	WBC 0 Protein 168	–	IVIG: no, PSL: partial but relapsed
P3 (CASPR1)	36/M	Acute	3	0	Sensory ataxia, distal weakness, tremor, neuropathic pain	DML: 2, F: 1, NCV: 1, DUR: 1, CB: 1, TD: 1,	WBC 23 Protein 562	–	IVIG: no
P4 (CNTN1)	68/M	Subacute	2	0	Superior oblique palsy followed by facial diplegia, sensory ataxia, distal weakness, tremor	DML: 2, F: 2, TD: 1, DUR: 1	WBC 6 Protein 101	Nephrotic syndrome diabetes	–
P5 (NF186/140)	48/M	Acute	297	3	Severe Quadriplegia, cranial nerve palsy, hypoesthesia	CB: 3, F: 3	WBC 0 Protein 49	Nephrotic syndrome (membranous glomerulonephritis on biopsy)	IVIG: no, PLEX: yes, PSL: partial but relapsed

P, patient; M, male; F, female; DML, distal motor latency; NCV, nerve conduction velocity; DUR, distal compound motor action potential duration; TD, temporal dispersion; CB, conduction block; IVIG, intravenous immunoglobulin; IVMP, intravenous methylprednisolone; PSL, oral prednisolone; PLEX, plasma exchange.

### 3.2 Clinical characteristics of AN patients

Table 1 summarizes clinical presentations, NCS and CSF findings, and responses to previous treatments of our patients. Four out of five patients were male, all but one with young age onset between their 20s and 40s. All patients received immunotherapy including MMF: three during their fourth episode (disease duration before treatment ranging from 12 to 297 months), and two during their first episode (2 to 3 months). Patients P1 to P4 presented with sensory ataxia, distal weakness, and hand tremor as their primary manifestation. P2 and P3 also experienced neuropathic pain. Isolated superior oblique palsy preceded the main peripheral nerve symptoms in P4, as previously reported (6). P5 (NF186/140) presented with severe quadriplegia (INCAT 10, MRC sum score 8), hypoesthesia, and cranial nerve involvement including dysphagia requiring enteric nutrition.

NCS findings are summarized in detail in Table 2. Patients P1 to P4 exhibited similar electrophysiological patterns, characterized by prolonged distal motor latency, prolonged or absent F-waves, slow nerve conduction velocity (NCV), prolonged distal CMAP duration, and rarely temporal dispersion or conduction block, indicating uniform conduction slowing. In contrast, P5 had numerous conduction blocks without temporal dispersion, as well as F-wave abnormalities. Notably, the conduction blocks resolved over the course of recovery without leaving any slowing, indicative of reversible conduction failure. All patient exhibited highly elevated CSF protein levels (101–562 mg/dL), except for P5 who had a normal CSF profile. Nephrotic syndrome was identified in P4 and P5, with the latter confirmed to have membranous glomerulonephritis through a kidney biopsy. In P3, enzyme-linked immunosorbent assay at nadir tested positive for GM1 IgG (53.1 EU/mL, normal range 0–20).

**Table 2:** Electrophysiological findings of the patients at diagnosis.

Parameter	P1	P2	P3	P4	P5	Normal limit
Median nerve						
DML (ms)	5.7*	10.2*	10.4*	**4.2**	**3.9**	3.6
MCV (m/s)	35*	28*	**NR**	52	**39**	50
dCMAP duration (ms)	9.0*	9.0*	8.9*	6.3	6.5	8.4^┼^
pCMAP duration (ms)	10.4	10.8	**NR**	6.7	6.1	
dCMAP amplitude (mV)	**1.6**	15.0	**0.7**	12.0	6.5	5
pCMAP amplitude (mV)	**1.6**	13.2	NR*	10.8	2.3*	5
F latency (ms)	47.7*	66.6*	**NR**	27.5	41.7*	25.7 ~ 32
SCV (m/s)	**32**	**NR**	**NR**	45	**41**	41
SNAP (μV)	**3.2**	**NR**	**NR**	**3.2**	**0.9**	10
Ulnar nerve						
DML (ms)	5.0*	5.9*	5.5*	3.9*	**3.3**	2.5
MCV (m/s)	29*	24*	14*	58	**36**	50.5
dCMAP duration (ms)	8.0	9.8*	8.4	6.3	8.0	9.6^┼^
pCMAP duration (ms)	10.4*	11.2	11.1*	6.8	7.8	
dCMAP amplitude (mV)	6.8	13.6	4.7	12.7	**2.6**	5
pCMAP amplitude (mV)	6.1	10.4	2.1*	10.9	0.4*	5
F latency (ms)	58.1*	NR*	39.9*	**30.6**	NR*	25.6 ~ 32.7
SCV (m/s)	**NR**	**NR**	**NR**	**NR**	**NR**	39.3
SNAP (μV)	**NR**	**NR**	**NR**	**NR**	**NR**	10
Tibial nerve						
DML (ms)	10.0*	**NR**	**NR**	**5.7**	**6.0**	5.1
MCV (m/s)	44	**NR**	**NR**	45	**40**	40.6
dCMAP duration (ms)	10.1*	**NR**	**NR**	5.9	5.8	9.2^┼^
pCMAP duration (ms)	11.8	**NR**	**NR**	6.4	6.7	
dCMAP amplitude (mV)	**1.5**	**NR**	**NR**	13.6	8.5	5
pCMAP amplitude (mV)	**1.0**	**NR**	**NR**	11.8	5.8	5
F latency (ms)	NR*	**NR**	**NR**	63.9*	61.8*	44.6 ~ 59.9
Peroneal nerve						
DML (ms)	**NR**	**NR**	**NR**	8.3*	**6.7**	4.8
MCV (m/s)	**NR**	**NR**	**NR**	58	**37**	50.5
dCMAP duration (ms)	**NR**	**NR**	**NR**	9.2*	6.9	8.8^┼^
pCMAP duration (ms)	**NR**	**NR**	**NR**	12.3*	6.7	
dCMAP amplitude (mV)	**NR**	**NR**	**NR**	**1.7**	**0.7**	4
pCMAP amplitude (mV)	**NR**	**NR**	**NR**	**1.2**	0.2*	4
F latency (ms)	**NR**	**NR**	**NR**	63.1*	**NR**	44.5 ~ 55.9
Sural nerve						
SNAP (μV)	22.1	**NR**	**NR**	17.0	**NR**	6
SCV (m/s)	41	**NR**	**NR**	41	**NR**	34.7

Abnormal values are marked in bold, and those indicative of demyelination according to the 2021 EAN/PNS guidelines are marked with an asterisk (*). The normative reference for distal CMAP duration was derived from the guideline, while references for other parameters were established based on healthy South Korean subjects. Note that normative values for F-wave latencies vary according to the subject’s height.

DML, distal motor latency; MCV, motor conduction velocity; CMAP, compound muscle action potential; p, proximal; d, distal; SCV, sensory conduction velocity; SNAP, sensory nerve action potential; NR, not elicited; EAN, European Academy of Neurology; PNS, Peripheral Nerve Society.

### 3.3 Treatment outcomes

**Figure 2:**
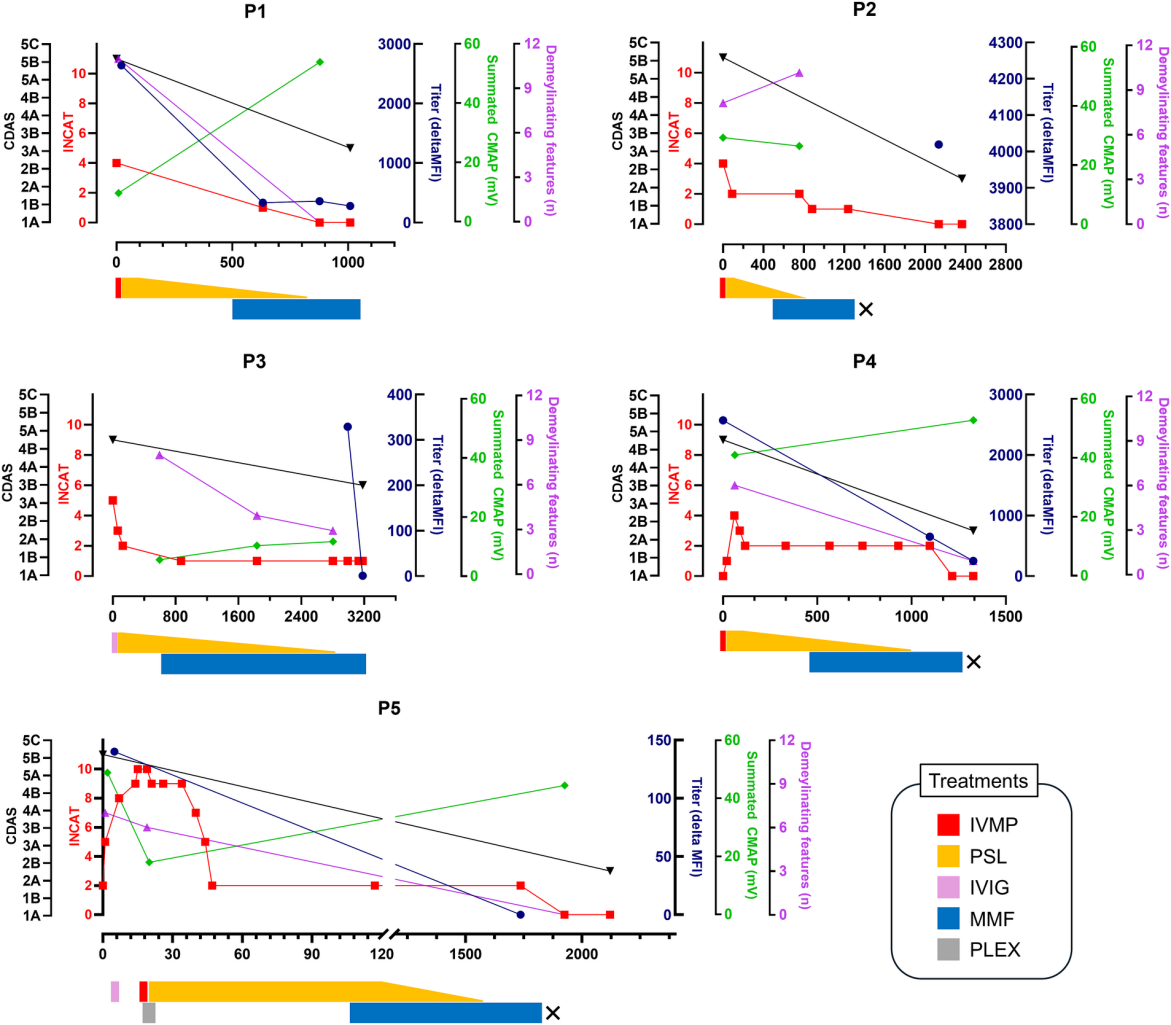
Clinical course of the patients. The timeline of clinical course, treatments, antibody titers, and electrophysiological changes according to days since symptom onset (x-axis) is presented. Clinical outcomes are shown using the INCAT disability score and CIDP disease activity status (CDAS). Treatment regimens are detailed below the clinical course, with “X” indicating discontinuation of MMF treatment. INCAT, inflammatory neuropathy cause and treatment; CDAS, CIDP disease activity status; MFI, median fluorescence intensity; CMAP, compound muscle action potential; IVMP, intravenous methylprednisolone; IVIG, intravenous immunoglobulin; PSL, prednisolone; MMF, mycophenolate mofetil; PLEX, plasma exchange.

Patients P1, P2, and P5 received seven lines of immunotherapy due to previous episodes. IVIG was ineffective for all, corticosteroids had a partial effect but led to relapses during maintenance. Plasma exchange was effective in P5. P3, who had an acute-onset first episode, initially received IVIG for a GBS diagnosis but was referred to our center due to a lack of response. Figure 2 summarizes the treatments administered at our center and the clinical courses for the last episode of each patient. All patients received MMF and PSL treatment following acute-phase treatments, primarily with IVMP. After treatment, all patients showed significant improvement in disability (INCAT 0 in all but one). No relapses or progression occurred during a median follow-up of 5.8 years (average 5.5 years). Despite long-term maintenance of MMF therapy (median of 2.3 years, average of 3.2 years), no adverse events requiring treatment cessation or dose reduction occurred. Three have been discontinued treatment with normal neurological examination (CDAS 2A in P2, P4, and P5), while the others are continuing MMF monotherapy. Significant reduction in antibody titers including negative conversion in P3 and P5 was noted in all patients with follow-up sera. In repeated NCS, all but one patients showed an increase in the summated CMAP amplitudes and a decrease in the number of demyelinating findings. Although the latter slightly increased in P2 at follow-up, this was related to the re-emergence of the previously non-elicitable CMAP in the peroneal nerve.

## 4 Discussion

We present clinical and paraclinical features of five patients with AN, focusing on their successful long-term outcomes following MMF and PSL therapy. As a novel and rare disease, there is insufficient evidence for the optimal treatment strategy for AN. IVIG has been found to be ineffective in many cases and may therefore be better avoided (7–10). Although rituximab shows promise, it may not be suitable as a first-line therapy given its high cost, lack of insurance coverage, and injection-related inconvenience. Perhaps for this reason in part, the ongoing clinical trial is only recruiting those who did not respond to first-line treatment (3). Moreover, it is also important to note that not all patients with AN respond to rituximab, partly due to persistent long-lived plasma cells or formation of anti-drug antibodies (11, 12). Risk of hypogammaglobulinemia and related infections also increases with repeated treatment (13, 14).

Our study suggests that MMF, being feasible, safe, and effective, has potential as a first-line maintenance therapy in AN when used subsequent to acute-phase treatments and with PSL. All our patients achieved complete or near-complete recovery from disability and effective control of disease activity after MMF and PSL treatment. Given that three of our patients had previously relapses several times despite PSL monotherapy, MMF likely have played a beneficial role. Since first approval in 1995, it is known that MMF is well-tolerated, with few severe complications, and is effective in many autoimmune disorders, including lupus, systemic sclerosis, myasthenia gravis, neuromyelitis optica, and myelin oligodendrocyte glycoprotein antibody disease (15–21). It inhibits inosine monophosphate dehydrogenase, blocks guanine synthesis via the *de novo* pathway, ultimately inhibiting autoreactive B and T cell growth and antibody production (22). Notably, its effectiveness in preventing relapses has been reported in leucine-rich glioma inactivated-1 antibody encephalitis, another IgG4 autoimmune disorder (23).

We performed comprehensive analyses of clinical outcomes as well as longitudinal changes in antibody titers and NCS. Beyond its diagnostic utility, measuring antibody titers in AN is also useful for monitoring treatment responses and predicting disease outcomes (1, 7, 10, 24–28). Significant reduction in autoantibodies in our patients supports the good clinical responses to treatment. A recent Japanese study reported that electrophysiological changes may also serve as an indicator of clinical fluctuations in patients with anti-NF155 AN (29). With this in mind, we analyzed NCS parameters, including summated CMAP amplitude and the number of demyelinating findings, confirming their correlation with clinical outcomes. These two indicators are known correlates with outcomes in CIDP and anti-MAG neuropathy (30–32).

Our study also provides valuable data on the frequency of AN among patients with a CIDP diagnosis based on a prospective cohort of 106 consecutive patients. Our result (4.7%) is consistent with previous large-scale studies from Italy (14 out of 276 patients, 5.1%), France-Belgium-Switzerland (27/1500, 1.8%), and Netherlands (12/181, 6.6%) (8, 9, 26). However, our results showed a lower prevalence compared to Asian studies, which reported higher prevalence rates suggesting potential ethnic differences. Several reasons might explain this discrepancy (33–36); most of these studies used retrospective cohorts and are thus prone to selection bias. Also, they included a relatively small number of patients. The variable performance of antibody assays depending on the methods used should also be considered. We primarily used live cell-based flow cytometry assay and confirmed results with a second technique as recommended by the guidelines to avoid false positives (4). As some of our patients were already under immunotherapy at the time of sampling, we acknowledge the possibility that the prevalence might have been underestimated.

The clinical and serological characteristics of patient P5 are noteworthy. He presented with relapsing fulminant GBS, concomitant nephrotic syndrome, and normal CSF protein, closely resembling AN associated with pan-NF antibodies (24, 37). However, this patient tested negative for NF155 and showed specific binding to NF186/140, which is a very rare case to our knowledge (38). He exhibited numerous conduction blocks, which completely resolved without leaving any slowing, as seen in axonal GBS, while the other patients with paranodal antibodies had relatively homogenous conduction slowing (39). Further studies are needed to address the heterogeneity in pathogenesis, electrophysiology, and its evolution pattern within AN spectrum. Moreover, epitope mapping of pan-NF, NF155, and NF186/140 autoantibodies would also be of interest.

Our study is limited by the small number of AN patients, the lack of controls treated with other maintenance therapy regimens. Heterogeneous acute-phase treatments and varying disease duration likely contributed to the outcomes. However, considering that our patients did not respond to IVIG and experienced multiple relapses with PSL monotherapy, and given the extreme rarity of this disease, we believe that potential utility of MMF warrants further investigation. Studies involving a larger cohort, with established treatment protocols and valid outcome measures to compare the efficacy, safety, and tolerability of different immunotherapies will be crucial for developing treatment guidelines for AN.

## Data Availability

The raw data supporting the conclusions of this article will be made available by the authors, without undue reservation.
