# Supplementary material for: Favorable long-term outcomes of autoimmune nodopathy with mycophenolate mofetil
Source: Front Neurol. 2024 Dec 12;15:1515161. doi: 10.3389/fneur.2024.1515161 (PMC11669590; doi:10.3389/fneur.2024.1515161)
Supplement: Supplementary file 1 [file Table_1.DOCX]

Supplementary Material

# Supplementary Figure 1. Results of flow cytometry assay to detect anti-NF140 IgG. Among the tested sera, only P5 showed specific binding to NF140-transfected cells (blue) compared to non-transfected cells (dark purple).


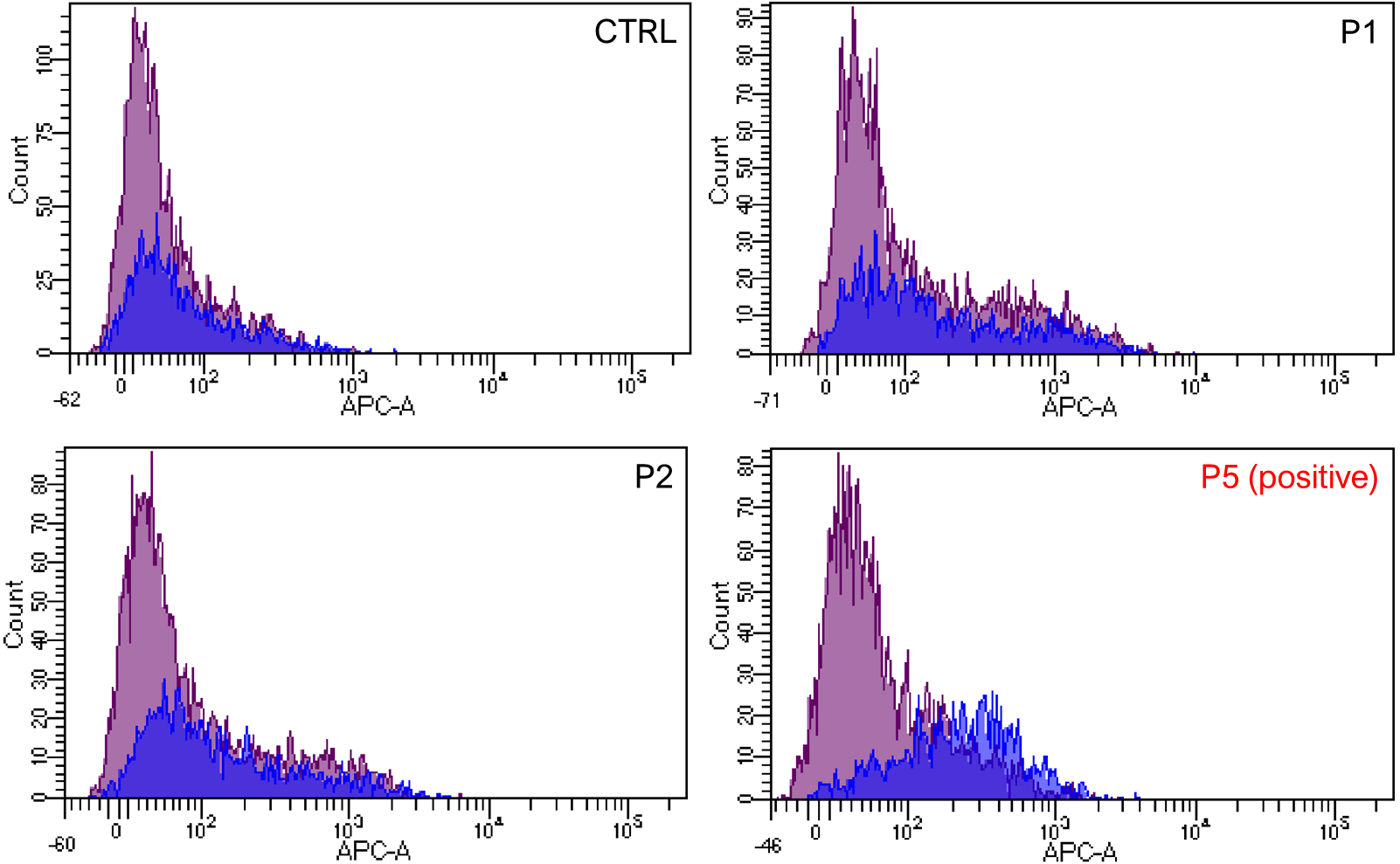


Abbreviations: NF140, neurofascin-140; CTRL, control; APC, Allophycocyanin.
